# Maspin, Syndecan-1, and Ki-67 in the Odontogenic Keratocyst: An Immunohistochemical Analysis

**DOI:** 10.1155/2020/7041520

**Published:** 2020-07-14

**Authors:** Huda M. Hammad, Omar M. Nagrash, Rima A. Safadi

**Affiliations:** ^1^Department of Oral Medicine and Oral Surgery, Faculty of Dentistry, Jordan University of Science and Technology, P. O. Box 3030, Irbid 22110, Jordan; ^2^Al-Ramtha Governmental Hospital, Ministry of Health, P. O. Box 132, Al-Ramtha 21410, Jordan

## Abstract

The odontogenic keratocyst (OKC) is a controversial lesion that was reclassified as a tumor with the name “keratocystic odontogenic tumor” in 2005. The reclassification was revoked recently in 2017, with a conclusion on the need for further studies on the subject. In this study, the expressions of an important regulatory protein (maspin), an important integral membrane proteoglycan (syndecan-1), and a universal proliferation marker (Ki-67) in the epithelium of the OKC were investigated in comparison with the dentigerous cyst (DC) and ameloblastoma (AB). Twenty-six OKCs, eleven DCs, and ten conventional ABs were immunohistochemically stained for maspin, syndecan-1, and Ki-67. ImageJ was used to analyze the positivity of maspin and syndecan-1. The Ki-67 score was calculated as the percentage of positive nuclei in 5 high power fields. Analysis of variance (ANOVA) test and Student *t*-test were used as appropriate. Lower expressions of maspin were noted in OKC and DC compared to those in AB, and lower expressions of syndecan-1 were noted in OKC and AB compared to those in DC. The differences, however, did not reach statistical significance (ANOVA and *t*-test: *P* > 0.05). The Ki-67 score was significantly higher in OKC than in DC (*t*-test: *P* < 0.05), and not significantly different from AB (*t*-test: *P* > 0.05). In conclusion, expressions of maspin and syndecan-1 are not strongly representative of differences in behavior between OKC, AB, and DC. However, the expression of Ki-67 indicates comparable proliferative activities of OKC and AB, which are higher than that of DC. Further investigation on the biologic behavior of OKC is still recommended to arrive at more specific conclusions regarding its classification.

## 1. Introduction

Odontogenic keratocysts (OKCs) comprise a significant proportion of odontogenic cysts. They are known to show locally aggressive behavior with a tendency to recur following excision. OKCs occur in both jaws, with a predilection to affect the posterior body, angle, and ramus area of the mandible. OKCs may occur as solitary or multiple lesions, and multiple OKCs may be associated with the nevoid basal cell carcinoma syndrome [1].

The OKC was reclassified as a tumor with the name “keratocystic odontogenic tumor” in 2005 [2]. Despite the accumulation of research work supporting this reclassification, the debate on whether to consider it as a tumor or cyst did not stop [1]. Moreover, the term “OKC” continued to be used by the scientific community more favorably than “keratocystic odontogenic tumor” [3]. The debate has culminated recently in the revocation of the reclassification in the 2017 WHO classification of diseases [4].

Various immunohistochemical studies have been conducted to investigate the biologic nature of OKCs, such as those assessing proliferation markers (Ki-67 and PCNA) [5–19] and the tumor suppressor gene P53 and other members of the P53 family [8, 12, 14, 15, 17, 18, 20–25]. Higher proliferative activity and more significant or different P53 expression in the OKC compared to other odontogenic cysts have been reported [5–9, 12–14, 16–22, 24]. Additionally, mutations or abnormalities of the PTCH, P53, P16, and MCC tumor suppressor genes have been reported to be associated with the etiology of sporadic and syndromic OKCs [26–33].

Despite the higher proliferative activity in the OKC than in other odontogenic cysts [5–9, 12–14, 16–19], the clinical regression of some OKCs following marsupialization is known to occur [34]. This fact was among the causes for revocation of the 2005 classification, since regression is not a feature of neoplasia [35, 36], and therefore, more investigations of the biologic nature of OKCs were recommended.

Some biologic markers have not been investigated in the OKC yet, such as mammary serine protease inhibitor (maspin). Other markers were investigated only in a few studies, such as syndecan-1 [37–41].

Maspin can be detected in many normal tissues, mainly epithelial. It has been determined to function as a tumor suppressor by increasing cell adhesion and apoptosis and decreasing motility, angiogenesis, and pericellular proteolysis [42]. Its expression may be either down- or upregulated in several benign and malignant tumors, and thus, its expression is considered to have prognostic implications. Its therapeutic effects are also being investigated [43].

Syndecan-1, also known as CD138, is a member of the syndecan family, which are integral membrane heparan sulfate proteoglycans. It is essential in cell-cell and cell-matrix interactions [44, 45]. It is mainly expressed in epithelial cells and plasmacytes. Its immunoexpression is altered in many inflammatory, infectious, fibrotic, and neoplastic diseases. Certain molecular pathways in which syndecan-1 is involved are deregulated during carcinogenesis. These pathways are related to cell proliferation, angiogenesis, apoptosis, and tumor invasion [45]. The altered immunoexpression in various types of cancer is sometimes correlated with patients' prognosis and clinicopathologic parameters [44, 45]. Due to its important role in carcinogenesis, syndecan-1 is a promising target for anticancer therapy [45].

This study aimed to continue the investigation of the biological nature of the OKC and shed more light on the “tumorous” versus “cystic” nature of this lesion. The expression and distribution of maspin and syndecan-1 were immunohistochemically investigated in the OKC and compared to those in the dentigerous cyst (DC) and ameloblastoma (AB). The expression of Ki-67 was also studied to be used as a baseline reference since its expression is already established in the literature [5, 7, 8, 11–13, 15–19].

## 2. Materials and Methods

All necessary approvals were obtained from the Deanship of Research, Jordan University of Science and Technology, including the Institutional Review Board approval.

### 2.1. Tissue Samples

The archives of the biopsy service of the Department of Pathology and Microbiology, Faculty of Medicine, Jordan University of Science and Technology, and the Pathology Laboratory at the King Abdullah University Hospital were reviewed for biopsies diagnosed as OKC, DC, and AB during twenty years. Inclusion criteria for OKC and DC were minimal or no inflammation and well-oriented tissue sections. For AB, inclusion criteria were conventional type and well-oriented tissue sections. An experienced oral pathologist evaluated the retrieved sections, and 26 OKCs, 11 DCs, and 10 ABs were included in the study.

### 2.2. Immunohistochemical Procedure

Three 4 *µ*m thick sections were cut from each tissue block and mounted on positively charged glass microscopic slides (Superfrost Plus microscope slides, 060SFP, DiaPath, Martinengo, Italy) coated with VECTABOND™ Reagent (SP-1800, Vector Laboratories, Inc., Burlingame, CA, USA). The three sections were used for immunohistochemical staining for maspin, syndecan-1, and Ki-67. An additional section was cut from each specimen for routine H&E staining. More sections were cut from one of the OKC specimens to be used as a negative control in each immunohistochemical run.

The immunohistochemical staining was performed using an automatic stainer (Dako Autostainer Plus, DakoCytomation Denmark, Glostrup, Denmark). The standard procedure using the EnVision™ + Dual Link System-HRP, Code K4061 (Dako, Glostrup, Denmark) was used, followed by Liquid DAB + substrate-chromogen system, code K3468 (Dako, Glostrup, Denmark). Sections were then counterstained with Mayer's hematoxylin.

The primary antibodies used were: rabbit anti-maspin polyclonal antibody, Maspin H-130: sc-22762, dilution 1 : 150 (Santa Cruz Biotechnology, Inc., Santa Cruz, California, USA), monoclonal mouse anti-human CD138, clone MI15, code M 7228, dilution 1 : 100 (Dako, Glostrup, Denmark), and monoclonal mouse anti-human Ki-67 antigen, clone MIB-1, code IS626, ready to use (Dako, Glostrup, Denmark).

In negative control sections for each run, the primary antibody was replaced by nonspecific mouse immunoglobulin G at 1 : 150 dilution (Biogenex Laboratories Inc., San Ramon, CA, USA). Occasional plasma cells found in some sections in each run served as internal positive controls for syndecan-1. Normal breast tissue was used as a positive control for maspin, and a case or oral squamous cell carcinoma was used as a positive control for Ki-67.

### 2.3. Microscopic Evaluation and Imaging

Digital images of two fields at ×400 magnification were acquired from each section stained with maspin or syndecan-1, using an Olympus DP20-5 digital camera mounted on an Olympus BX50 light microscope (Olympus Corporation, Tokyo, Japan). The most representative field was selected for image analysis. For Ki-67 stained sections, images of five high power fields (HPFs) at ×400 magnification were acquired to allow calculation of the Ki-67 score.

### 2.4. Image Analysis

Digital images of tissue sections stained for maspin and syndecan-1 were prepared for analysis using the ImageJ 1.44 program. ImageJ is a public domain Java image processing program developed at the National Institute of Health (Bethesda, Maryland, USA). The Color Deconvolution Plug-in was downloaded into the ImageJ program and used to isolate positively stained areas from the total epithelial area as previously described [46]. For Ki-67 stained sections, the percentage of Ki-67 positive cells in 5 HPFs for each case was determined and expressed as the Ki-67 score [47, 48].

### 2.5. Statistical Analysis

Data for each diagnostic group were entered into the Statistical Package for the Social Sciences (SPSS) version 15 (SPSS Inc., Chicago, IL, USA). The percentage of the positive area was calculated by dividing the positive area by the total epithelial area for each case. The mean proportions between the different groups in the study were compared using parametric tests. The significance of immunohistochemical positivity as a factor in separating the diagnostic groups was calculated using the ANOVA test. Pairwise comparisons were performed using the Student *t*-test. The significant *P*-value was considered to be less than 0.05.

## 3. Results

### 3.1. Microscopic Findings

Negative and positive control sections included in each run were evaluated before analyzing the study tissue sections. All negative control sections consistently showed a lack of brown staining. Breast tissue used as a positive control for maspin showed strong expression in myoepithelial cells and weaker focal expression in glandular epithelial cells. Occasional plasma cells used as an internal positive control for syndecan-1 showed strong membranous expression. Squamous cell carcinoma control tissue for Ki-67 showed nuclear staining of scattered cells.

**Figure 1:**
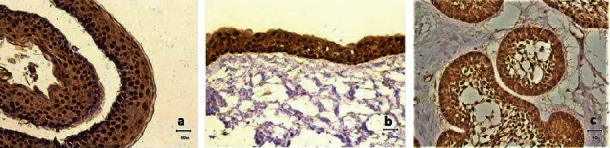
Immunohistochemical expression of maspin (a) in OKC epithelial lining, (b) in DC epithelial lining, and (c) in AB (original magnification ×400).

All the OKCs were positive for maspin. The pattern of expression in the epithelium was both nuclear and cytoplasmic. The distribution involved the full epithelial thickness (Figure 1(a)). The expression of maspin in the epithelial lining of DCs was also cytoplasmic and nuclear. The distribution of maspin-positive cells involved the entire thickness of the epithelium (Figure 1(b)). All cells in AB sections showed positivity, but the peripheral columnar or cuboidal cells showed a stronger reaction than the stellate reticulum-like cells (Figure 1(c)).

**Figure 2:**
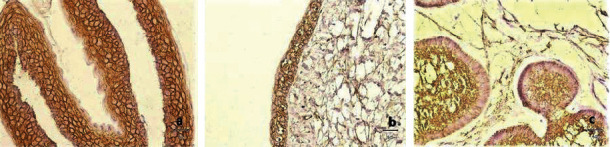
Immunohistochemical expression of syndecan-1 (a) in OKC epithelial lining, (b) in DC epithelial lining, and (c) in AB (original magnification ×400).

The expression of syndecan-1 in OKCs was localized to the membranes of epithelial cells and intercellular junctions in the entire thickness of the epithelium. The parakeratinized layer showed negative staining (Figure 2(a)). Similarly, syndecan-1 expression in DCs was localized to the cell membranes of epithelial cells (Figure 2(b)). All cases of AB showed positive immunoreactions with syndecan-1. Stellate reticulum-like cells reacted with stronger intensity than the peripheral columnar or cuboidal cells (Figure 2(c)). When present, areas of squamous metaplasia were not reactive.

**Figure 3:**
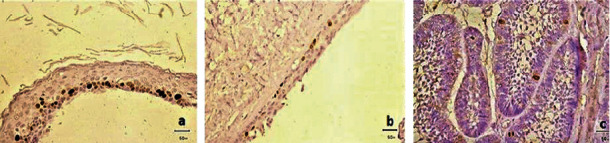
Immunohistochemical expression of Ki-67 (a) in OKC epithelial lining, (b) in DC epithelial lining, and (c) in AB (original magnification ×400).

The expression of Ki-67 protein in OKCs was exclusively localized to the nuclei of the basal and suprabasal cell layers of the epithelium. Nuclear expression was predominantly suprabasal, but occasional positive basal nuclei were present (Figure 3(a)). Occasional Ki-67 positive nuclei in DCs were primarily distributed in the basal cell layer (Figure 3(b)). All cases of AB showed positive nuclear staining with Ki-67. Positive nuclei were predominant in the peripheral columnar and cuboidal cells, but stellate reticulum-like cells occasionally expressed Ki-67 positive nuclei (Figure 3(c)).

### 3.2. Image Analysis

Table 1 shows the frequency distribution of the mean, standard deviation, minimum, and maximum values of the percentages of maspin and syndecan-1 positive areas, and the Ki-67 scores in the three diagnostic groups. The mean percentages of maspin-positive areas were close for the DC and OKC. The mean percentage for AB was the highest. The mean percentage of syndecan-1 positive areas had close values for the three groups. The mean Ki-67 scores were close for the OKC and the AB, and much lower for the DC.

Table 2 shows the results of multiple comparisons of the mean percentages of maspin and syndecan-1 positive areas, and the Ki-67 score in the three diagnostic groups. The mean percentages of both maspin and syndecan-1 positive areas in the three diagnostic groups were not significantly different (ANOVA:*P*-value > 0.05). Meanwhile, the Ki-67 scores were significantly different (ANOVA: *P*-value < 0.05) between the three groups.

Table 3 shows the results of paired comparisons of the mean percentages of maspin and syndecan-1 positive areas, and the Ki-67 scores in the OKC with that in the DC and AB. The mean percentages of both maspin and syndecan-1 positive areas in the OKC were not significantly different from those in the DC nor the AB (independent samples *t*-test: *P*-value > 0.05). The mean Ki-67 score in the OKC was significantly higher than that in the DC (independent samples *t*-test: *P*-value < 0.05) and not significantly different from that in the AB (independent samples *t*-test: *P*-value > 0.05).

## 4. Discussion

The WHO reclassification of the OKC in 2005 as a benign odontogenic tumor rather than an odontogenic cyst was a result of its locally aggressive behavior and relatively high recurrence rate, in addition to the studies on genetic and molecular mechanisms involved in its development and progression [5–11, 20–22, 26–31]. The debate has culminated recently in the revocation of the reclassification in the 2017 WHO classification of diseases [4]. The consensus panel considered evidence of a neoplastic nature of OKC to be currently lacking or insufficient and concluded that further research on the subject is needed [35, 36].

In this study, maspin was investigated for the first time in the OKC to shed more light on the biologic aspect and add to the immunohistochemical profile of this “controversial” lesion. Maspin was found to be expressed in the entire epithelial thickness of all cases of OKC. The expression was similar in pattern among all study groups. The distribution was both cytoplasmic and nuclear, which is consistent with the pattern reported by Vered et al. [49]. In their study, they compared maspin expression in central low-grade mucoepidermoid carcinoma and glandular odontogenic cyst. They used radicular and dentigerous cyst in which the epithelial lining showed mucous metaplasia as a control group. Cyst lining in all the cysts in the study showed positive immunohistochemical staining for maspin. The pattern was diffuse in both cytoplasm and nuclei of epithelial cells, but not in mucous cells, which were negative.

In this study, the pattern of expression of maspin in AB was consistent with the findings of Kumamoto and Ooya [50], who reported stronger maspin reactivity in peripheral columnar or cuboidal cells than in central cells. Maspin expression has not been investigated in the OKC before. However, the abovementioned study compared maspin expression in tooth germs, ABs, and malignant ameloblastic tumors (metastasizing ABs and ameloblastic carcinomas) [50]. The expression was significantly higher in ABs than in tooth germs, metastasizing ABs showed strong expression, while ameloblastic carcinomas showed weak expression in some cells. Similarly, maspin expression in this study was highest in AB compared to DC and OKC, albeit statistically not significant. Therefore, it can be inferred that maspin expression increases from normal odontogenic structures to odontogenic cysts (DC and OKC), to the more locally aggressive tumor (AB).

The regulation of expression of maspin, however, is a complex process, influenced by P53 and other members of the family, namely, P63 and P73 [51–53]. Studies have shown different patterns of expression of the P53 family proteins in the OKC compared to other odontogenic cysts [8, 12, 14, 15, 17–25]. This finding warrants additional research on maspin expression in the OKC concerning the P53 gene family members.

Syndecan-1 showed no significant differences between the three study groups, although the expression was lower in AB and OKC compared to DC. Syndecan-1 participates in cell proliferation, cell migration, intercellular adhesion, cell-matrix interactions, and cytoskeleton organization. Intercellular adhesion and attachment with the extracellular matrix are affected by the loss of its expression [44]. Downregulation of syndecan-1 expression has been reported in epithelial dysplasia and oral carcinoma [44, 54, 55]. It has been associated with poorer prognosis of oral carcinoma and increased aggressiveness of ameloblastoma [44, 47]. The lower syndecan-1 expression in the OKC compared with DC might explain its local aggressiveness and increased potential for recurrence in comparison with the clinical behavior of the DC. However, since the difference is not significant, it may be that its effect on aggressive behavior in the studied lesions is limited.

The results of this study are consistent with those of Nadalin et al., who found no significant difference in the expression of syndecan-1 between the DC and OKC [37]. They are also consistent with those of Etemad-Moghadam and Alaeddini [40], who compared syndecan-1 expression in different odontogenic lesions, including AB and OKC. They found that only odontogenic myxomas showed a significant difference from other lesions since they showed no expression.

The results are partially different from those of Al-Otaibi et al., and different from those of Al-Otaibi et al. [38, 41]. Brito-Mendoza et al. found no significant difference between OKC and DC but found the mean rank scores of AB to be significantly lower than those of OKC and DC [38]. The difference between this study and their study may be explained by the difference in the methodology used. While they applied semiquantitative analysis, we utilized a computerized image analysis system. It could also be explained by the variation in the degree of inflammation between lesions. Unlike this study, there is no mention in their research on the exclusion of markedly inflamed cases. It is worthwhile to mention here that Nadalin et al. observed low or absent expression of syndecan-1 in areas of OKC lining in which there was an alteration in epithelial morphology due to inflammation [37].

The distribution patterns of syndecan-1 in the epithelia of DC, OKC, and AB in this study were consistent with the findings of other studies [37, 38, 40, 41]. They were, however, unlike those of Özcan et al., who reported a different distribution in the OKC, describing it as a strong membranous expression in basal and suprabasal cells only, while, as in this study, it involved the full thickness of DC lining [39].

As for the Ki-67, positive nuclei were significantly higher in the OKC epithelial lining than that of the DC. The Ki-67 positive nuclei were predominantly in the suprabasal cell layer of the OKC with occasional ones in the basal cell layer, while they were mainly in the basal cell layer of the DC. The results of this study indicate that OKC has an intrinsic proliferative activity comparable to that of AB but significantly higher than that of DC. The findings of this study are consistent with those of other studies comparing the Ki-67 expression in the OKC to other odontogenic cysts. Those studies showed that the suprabasal cell layers in the epithelial lining of the OKC have higher proliferative activity when compared with the basal cell layer and when compared with the linings of other odontogenic cysts [5, 7, 8, 11–13, 16–19]. Some studies that compared the Ki-67 expression in the OKC with that in the AB reported a significantly higher proliferation index in the OKC than in AB [18, 48, 56, 57], while one study reported the opposite, a significantly higher proliferation index in AB than in the OKC [58]. Similar to the results of this study, Thosaporn et al. reported a slightly higher but not significantly different proliferation index in AB than in OKC, using the IPO-38 antigen [59]. The difference from the studies which indicated a considerably higher proliferation index in the OKC than in AB might be explained by the strict criteria that were applied in selecting the specimens in this study. All sections showing considerable inflammation were excluded, ending up with only 47 specimens out of 200. Indeed, one study indicated a statistically significant increase in the expression of Ki-67 in the lining of inflamed OKCs compared to noninflamed ones [60]. Another study suggested that inflammation has no significant effect on the overall proliferation activity of OKC. However, a focal increase in Ki-67 expression adjacent to moderately to severely inflamed areas was found [61].

Since one of the main purposes of this study was to compare the OKC to a more aggressive odontogenic lesion, the conventional AB was chosen. Unicystic AB would not have served this purpose since the latter is known to have less aggressive clinical and biological behavior than conventional AB [62]. In this regard, it has been shown that the expression of syndecan-1 is higher in unicystic AB than in solid/multicystic or conventional AB [38, 47, 63], and using it would affect the statistical significance of the results. However, it would be worthwhile in future studies to consider investigating additional biologic markers in different histologic variants of AB, including the unicystic.

## 5. Conclusions

The expressions of maspin and syndecan-1 do not powerfully represent differences in biologic behavior between OKC, conventional AB, and DC. The expression of Ki-67, however, indicates comparable proliferative activities of OKC and conventional AB, which are higher than that of DC.

Additional research on the biologic behavior of OKC, considering more biologic markers and different control groups, is recommended to arrive at more specific conclusions regarding its classification.

## Figures and Tables

**Table 1:** Frequency distribution of the mean, standard deviation (SD), minimum, and maximum values of the percentages of maspin-positive areas, syndecan-1 positive areas, and the Ki-67 scores in the three study groups.

Study group	Marker	*N* ^*∗*^	Mean positive area percentage	SD	Minimum percentage	Maximum percentage
OKC	Maspin	26	52.54%	0.108	33%	69%
DC		11	53.36%	0.099	41%	66%
AB		10	60.40%	0.163	35%	81%

OKC	Syndecan-1	26	39.58%	0.13	12%	63%
DC		11	43.18%	0.18	11%	81%
AB		10	39.80%	0.15	12%	63%

OKC	Ki-67	26	23^*∗∗*^	11.17	8	49
DC		11	5^*∗∗*^	3.10	0	10
AB		10	24^*∗∗*^	20.65	0	62

^*∗*^Number of specimens. ^*∗∗*^Mean Ki-67 score.

**Table 2:** Multiple comparisons of the mean percentages of maspin-positive areas, syndecan-1 positive areas, and the Ki-67 scores in the three diagnostic groups, using ANOVA test. A significant *P*-value is shown in bold font.

Study group	Marker	*N* ^*∗*^	Mean positive area percentage	*F*	*P*
OKC	Maspin	26	52.54%	1.62	0.21
DC		11	53.36%		
AB		10	60.40%		

OKC	Syndecan-1	26	39.58%	0.385	0.68
DC		11	43.18%		
AB		10	39.80%		

OKC	Ki-67	26	23^*∗∗*^	9.52	**0.000**
DC		11	5^*∗∗*^		
AB		10	24^*∗∗*^		

^*∗*^Number of specimens. ^*∗∗*^Mean Ki-67 score.

**Table 3:** Paired comparisons of the mean percentages of maspin and syndecan-1 positive areas, and Ki-67 scores in the OKC with that in the DC and AB, using the independent samples *t*-test. A significant *P*-value is shown in bold font.

Study group	Marker	*N* ^*∗*^	Mean positive area percentage	*T*	Mean difference	*P*
OKC	Maspin	26	52.54%	−0.22	−0.008	0.8
DC		11	53.36%			

OKC	Syndecan-1	26	39.58%	−0.88	−0.046	0.4
DC		11	43.18%			

OKC	Ki-67	26	23^*∗∗*^	5.4	18.7	**<0.0001**
DC		11	5^*∗∗*^			

OKC	Maspin	26	52.54%	−1.7	−0.079	0.1
AB		10	60.40%			

OKC	Syndecan-1	26	39.58%	−0.245	−0.012	0.8
AB		10	39.80%			

OKC	Ki-67	26	23^*∗∗*^	−0.2	−1.05	0.8
AB		10	24^*∗∗*^			

^*∗*^Number of specimens. ^*∗∗*^Mean Ki-67 score.

## Data Availability

The statistical data used to support the findings of this study are included within the article.
